# Transcriptional regulators of human oncoviruses: structural and functional implications for anticancer therapy

**DOI:** 10.1093/narcan/zcac005

**Published:** 2022-03-03

**Authors:** Ivona Nečasová, Martin Stojaspal, Edita Motyčáková, Tomáš Brom, Tomáš Janovič, Ctirad Hofr

**Affiliations:** Institute of Biophysics of the Czech Academy of Sciences, Scientific Incubator, Královopolská 135, Brno 612 65, Czech Republic; LifeB, Functional Genomics and Proteomics, National Centre for Biomolecular Research, Faculty of Science, Masaryk University, Kamenice 753/5, Brno 625 00, Czech Republic; Institute of Biophysics of the Czech Academy of Sciences, Scientific Incubator, Královopolská 135, Brno 612 65, Czech Republic; LifeB, Functional Genomics and Proteomics, National Centre for Biomolecular Research, Faculty of Science, Masaryk University, Kamenice 753/5, Brno 625 00, Czech Republic; Institute of Biophysics of the Czech Academy of Sciences, Scientific Incubator, Královopolská 135, Brno 612 65, Czech Republic; LifeB, Functional Genomics and Proteomics, National Centre for Biomolecular Research, Faculty of Science, Masaryk University, Kamenice 753/5, Brno 625 00, Czech Republic; LifeB, Functional Genomics and Proteomics, National Centre for Biomolecular Research, Faculty of Science, Masaryk University, Kamenice 753/5, Brno 625 00, Czech Republic; LifeB, Functional Genomics and Proteomics, National Centre for Biomolecular Research, Faculty of Science, Masaryk University, Kamenice 753/5, Brno 625 00, Czech Republic; Institute of Biophysics of the Czech Academy of Sciences, Scientific Incubator, Královopolská 135, Brno 612 65, Czech Republic; LifeB, Functional Genomics and Proteomics, National Centre for Biomolecular Research, Faculty of Science, Masaryk University, Kamenice 753/5, Brno 625 00, Czech Republic

## Abstract

Transcription is often the first biosynthetic event of viral infection. Viruses produce preferentially viral transcriptional regulators (vTRs) essential for expressing viral genes and regulating essential host cell proteins to enable viral genome replication. As vTRs are unique viral proteins that promote the transcription of viral nucleic acid, vTRs interact with host proteins to suppress detection and immune reactions to viral infection. Thus, vTRs are promising therapeutic targets that are sequentially and structurally distinct from host cell proteins. Here, we review vTRs of three human oncoviruses: HBx of hepatitis B virus, HBZ of human T-lymphotropic virus type 1, and Rta of Epstein–Barr virus. We present three cunningly exciting and dangerous transcription strategies that make viral infections so efficient. We use available structural and functional knowledge to critically examine the potential of vTRs as new antiviral-anticancer therapy targets. For each oncovirus, we describe (i) the strategy of viral genome transcription; (ii) vTRs’ structure and binding partners essential for transcription regulation; and (iii) advantages and challenges of vTR targeting in antiviral therapies. We discuss the implications of vTR regulation for oncogenesis and perspectives on developing novel antiviral and anticancer strategies.

## Graphical Abstract

**Graphical Abstract:**
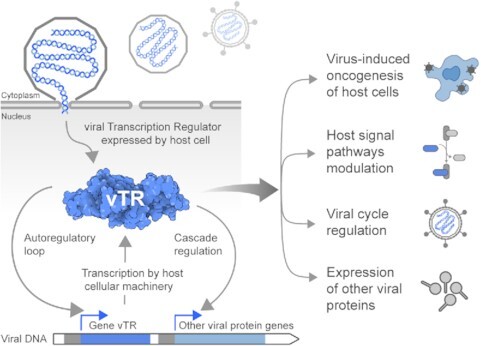
Transcriptional regulators of human oncoviruses.

## INTRODUCTION

Virus-associated diseases are the most challenging threats to humankind. In the last few decades, effective strategies to combat viral diseases have been developed and implemented worldwide. We have been able to eliminate several viral pathogens by vaccinations. An example of an effective elimination strategy is the successful eradication of smallpox by the World Health Organization (1), with the significant contribution of scientists and medical experts from the whole world (2,3). Unfortunately, there are viral diseases against which suitable vaccine approaches are insufficient, and we have to treat patients with ongoing infection. Several viruses are associated with cancer in humans. In 2008, there were an estimated 12.7 million new cases of cancers that accounted for 7.6 million deaths globally (4–6). One-sixth of cancer cases worldwide is linked to infectious agents, including viruses, bacteria and parasites (7). Oncoviruses cause 12% of all cancers (8) that account for >1.5 million new cases estimated per year.

Human oncoviruses comprise seven viruses: hepatitis B and C viruses (HBV and HCV), human papillomavirus (HPV), Epstein–Barr virus (EBV), human T-cell lymphotropic virus 1 (HTLV-1), Merkel cell polyomavirus (MCPyV) and human herpesvirus-8, also called Kaposi’s sarcoma-associated herpesvirus (KSHV) (6). On hand hand, human oncoviruses HPV, EBV, HTLV-1, MCPyV and KSHV contribute to oncogenesis directly via the translation of viral nucleic acid followed by viral protein expression. On the other hand, oncoviruses HBV and HCV contribute to the oncogenesis of host cells indirectly via chronic inflammation (9). Despite being DNA- and RNA-based viruses, oncoviruses share several common features: (i) Oncoviral infection is frequent, but the incidence of cancer development is rare –below 10 cases per 100 000. (ii) Oncoviruses that prevent early host cell lysis rather persist latently for a long time – up to several decades. Latent evasion strategy allows viruses to hide from the host immune response. Only a few specific cases, such as KSHV-induced Kaposi’s sarcoma and HPV-induced cervical cancer, can arise as tumors within months of infection. (iii) Oncoviruses cannot trigger tumorigenesis without the incidence of additional risk factors, such as immune suppression, chronic inflammation, co-infection with other pathogens and host mutations (10). Thus, virus-induced infection is just one component of the complex carcinogenesis process.

So far, there are no effective cures specifically targeting viral-induced malignancies in humans (10–12). The most common antiviral drugs are inhibitors of viral DNA polymerases: nucleoside analogs such as acyclovir and acyclic nucleoside phosphonates such as cidofovir, adefovir and tenofovir (13). Clinically used antiviral drugs are accompanied by nephrotoxicity and reversible neurological toxicity, as in the case of acyclovir (14). If nucleoside analogs are used in chronic patients, a high incidence of acquired resistance is observed (15).

The design of new antiviral drugs might be focused on viral proteins. Targeting viral proteins yields specific, less toxic compounds with a narrow spectrum of antiviral activity compared to targeting host cell proteins (16). Therapeutically targeted viral proteins must be essential for the survival of viruses and multiplication of viral nucleic acids. Viral transcription regulators (vTRs) fully meet the exclusivity requirement for specific antiviral therapy, as vTRs are critical proteins in viral genome transcription. The inhibition of vTRs interrupts viral DNA or RNA synthesis that is indispensable for the complete viral life cycle. Despite the apparent importance of vTRs in human health and their potential as therapeutic targets, little is known about vTRs' structure, binding specificities to nucleic acids, host target proteins, transcriptional cofactors and precise molecular mechanisms of their action.

### Roles of vTRs in the transcription of viral genomes: structural and functional properties of vTRs and research methodologies

**Figure 1.**
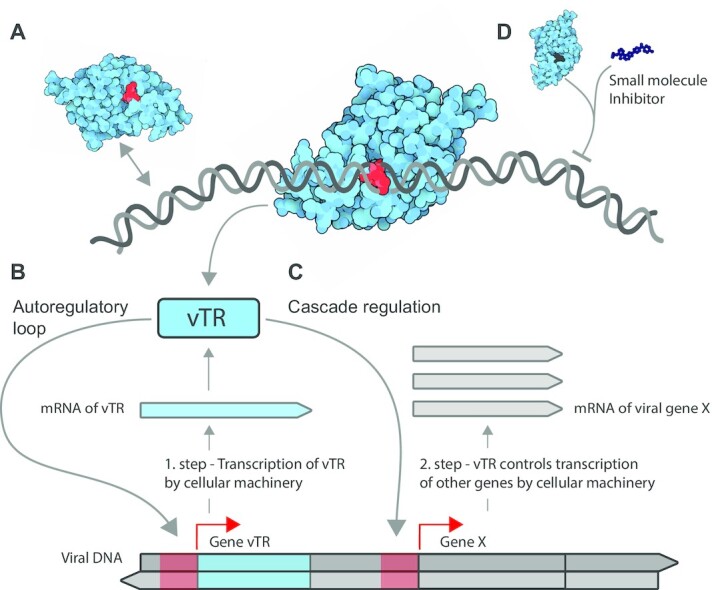
How vTRs stimulate transcription of viral genes. (**A**) vTRs act in concert with the host cellular transcriptional machinery components and control the processing of viral genetic information via direct binding to nucleic acids or through binding to host transcriptional cofactors. The scheme shows how vTRs hijack cellular transcriptional components to transcribe the viral gene encoding vTR. Once synthesized and returned to the nucleus, vTR stimulates transcription of the same transcription unit (vTR itself) via a two-step autoregulatory loop (**B**) or a different viral protein X via cascade regulation (**C**). Red arrows depict the end of promoters (red regions) on the genome where vTRs bind. (**D**) Identified regions essential for vTR function may serve to design small molecules that specifically inhibit vTRs, suppress viral genome transcription and activate host cell protective pathways.

In close cooperation with host transcriptional regulators, vTRs coordinate viral and human gene expression at several levels comprising transcription initiation, RNA polymerase recruitment, transcription elongation and chromatin organization. Accordingly, vTRs bind to nucleic acids either directly (Figure 1A) or indirectly through cofactors to modulate target gene expression. Additionally, vTRs modulate gene expression by targeting the transcriptional machinery or by altering chromatin states. The transcription machinery is eminently complex as viral genome transcription involves host cell pathways. Viral genes are usually transcribed in a particular temporal sequence. Tightly regulated transcription employs host cellular control mechanisms, such as signal transduction cascades that transmit specific environmental stimuli to the transcriptional machinery or cellular proteins that repress transcription. However, vTRs are critical components in establishing the order of viral gene transcription.

In general, to describe structure–function relationships of vTRs, a combination of biological (*in vivo*) and experimental (*in vitro*) approaches is required to find new structural motifs of vTRs that are essential for unique functions. To discover vTRs’ gene targets and determine their activation or repression activities, *in vivo* functional studies employing reporter and overexpression assays are carried out. The vTRs that affect transcription indirectly have been identified and characterized through protein–protein interactions with host transcription factors and cofactors using immunoprecipitations followed by mass spectrometry (17). Next, chromatin immunoprecipitation (ChIP) in virus-infected cells or cells transfected with a particular vTR is employed to detect vTRs that are associated with DNA. Previous binding studies between vTR and host genome used ChIP followed by high-throughput sequencing (ChIP-seq) (18).

After identification of vTRs’ binding targets, *in vitro* binding assays are performed for DNA–vTR and host protein–vTR complexes (19). To fully characterize DNA-binding and protein-binding specificities, a combination of methods such as electromobility shift assay (EMSA), fluorescence spectroscopy and isothermal titration calorimetry is employed (20,21). Analogically, *in vitro* binding assays establish the ability of vTRs to bind canonical DNA sequences and specific domains of host and viral proteins.

Recently, Liu *et al.* reviewed target genes, proteins and pathways regulated by particular vTRs and the relation of vTRs to human disease pathogenesis along with methodologies used for vTRs’ characterization. Out of the total of 419 vTRs, 53 have been connected to cancer development. So far, 22 oncogenic vTRs have been confirmed to interact directly with nucleic acids (18). Here, we focus on three vTRs that interact with different primary targets contributing to oncogenesis: (i) HBx (HBV X protein) binding double-stranded DNA (dsDNA) and long noncoding RNA (lncRNA) that is involved in HBV – causing cirrhosis and hepatocellular carcinoma; (ii) HBZ (HTLV-1 bZIP factor) regulating transcriptional machinery indirectly via cofactors upon HTLV-1 infection – causing adult T-cell lymphoma; and (iii) Rta (replication and transcription activator) binding dsDNA within EBV – causing infectious mononucleosis along with lymphoproliferative diseases. The following sections describe particular roles and the most critical interacting partners of vTRs mediating three diverse oncoviral transcription strategies.

### HBV: life cycle and strategy of viral genome transcription

**Figure 2.**
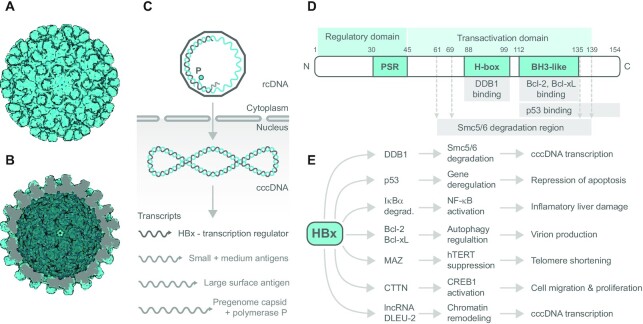
HBV capsid, viral DNA processing and HBx transcription factor domain structure, interactions and effects. The electron microscopy reconstruction of HBV capsid particle based on PDB 2G33: (**A**) outer view and (**B**) inner view, visualized using the Protein Imager. (**C**) Scheme of how HBV genome is processed in the host cell. Viral genome enters host cell as rcDNA (relaxed circular partially gapped dsDNA) containing viral reverse transcriptase (polymerase P, cyan circle) that converts rcDNA into dsDNA utilizing host RNA polymerase II and RNA primer at the 5′ end of the gapped strand (light gray); cccDNA (covalently closed circular DNA). (**D**) Domain structure of viral transcription factor HBx. Numbers above denote position of amino acid; PSR, proline/serine-rich region; H-box, amino acid sequence interacting with cellular DDB1; BH3-like, the region comprising BH3-like motif and interacting with Bcl-xL protein family. (**E**) Overview of cellular proteins directly interacting with HBx along with immediate effects of interaction at the cellular level, and subsequent physiological response. Other HBx interacting partners are mentioned in the text.

HBV is a hepadnavirus containing a DNA genome. HBV causes acute damage to infected hepatocytes. The continuous release of viral particles (Figure 2A and B) evokes an immune response that promotes liver inflammation. The prolonged inflammation induces severe liver damage, insufficiency and an elevated risk of cirrhosis and tumorigenesis (22).

From the molecular point of view, the HBV genome comprises 3.2 kb of relaxed circular partially gapped dsDNA (rcDNA) (Figure 2C). One DNA strand is full length, but the complementary (+) DNA strand is incomplete (23). The transcription mechanism is mediated by host cell proteins that repair the viral (+) DNA strand and translocate rcDNA to the nucleus. Repaired, stable and entirely complementary DNA called covalently closed circular DNA (cccDNA) is transcriptionally active. The (-) strand of cccDNA is the transcription template for cellular RNA polymerase II to produce a longer than genome length RNA called the pregenome RNA (pgRNA) and shorter transcripts, all of which serve as mRNAs (Figure 2C). The transcripts are exported to the cytosol for subsequent translation. The reverse transcription of pgRNA into the progeny DNA leads to the formation of rcDNA or the creation of linear dsDNA. The dsDNA mediates and significantly elevates the risk of HBV incorporation into the genome of hepatocytes (24,25). The shortest transcript encodes the HBx (Figure 2C). HBx is expressed early after the infection and rapidly promotes HBV genome transcription (26). HBx stimulates HBV transcription by activating cellular transcription pathways and affecting epigenetic signals on the cccDNA (27). HBV replication requires HBx, as confirmed in human hepatoma chimeric mice cells infected by HBV. In the experiment, HepaRG cells were infected with modified HBV expressing HBx under an inducible promoter. Other cells were infected by HBV only when HBx was expressed (28). Hence, HBx has been proven to be an essential regulator during the infection.

### HBx structure and binding partners: transcription regulation roles

HBx is a 154-amino acid, 17-kDa protein. As presented in Figure 2D, the N-terminal part contains a phylogenetically conserved region rich in proline and serine residues (PSR). The PSR exerts autoregulatory activity via phosphorylation-based signaling and alters the subcellular localization of HBx (29,30). HBx enhances active HBV metabolism (cccDNA transcription) by subverting DNA repair-associated ubiquitin ligase machinery comprising cullin 4–DNA damage-binding protein 1 (CUL4–DDB1) (31). HBx employs the amino acid sequence of H-box (Figure 2D) that resembles the DWD motif present in DDB1 cullin-associated factors. The structurally conserved DWD motif is formed by a short α-helix (32). HBx through its helical H-box interacts with DDB1 directly (33,34). The primary function of the complex HBx–DDB1–E3 ligase is to target the Smc5/6 complex for degradation, through which HBx prevents the Smc5/6 complex from restricting viral cccDNA transcription (Figure 2E). Site-directed mutagenesis verified that amino acids C61, C69, C137 and H139 are substantial for Smc5/6 degradation effects of HBx, although they are not essential for DDB1 binding (34). Recently, Kornyeyev *et al.* showed that wild-type HBx is predominantly located in the nucleus of HBV-infected primary human hepatocytes. In contrast, an HBx mutant that could not bind DDB1 (33) was detected in both the cytoplasm and nucleus (35).

HBV strategy on the onset of infection toward enhanced survival of infected hepatocytes relies on suppressing endogenous metabolic stress elicited by the presence of viral transcripts and their products (36). HBx interacts directly with tumor suppressor p53 through a region comprising amino acids 112-136. Upon HBx binding, the DNA binding site on p53 remains unaltered, and HBx downregulates activation of transcription mediated by p53 (37). Hence, HBx affects p53 transcriptional activity but has no effect on DNA binding of p53.

Besides inhibiting several p53-mediated cellular processes that include DNA sequence-specific binding, transcriptional transactivation and apoptosis (38), HBx indirectly influences cell survival by affecting NF-κB (nuclear factor of kappa B) pathways (39). NF-κB is a member of the family of transcription factors that play an essential role in immune, inflammatory and apoptotic responses. HBx binds directly NF-κB-associated inhibitor – IκBα. HBx interacts with amino acids 249-253 of IκBα. This region overlaps with the interaction site for p50 and p65 subunits of NF-κB, and it is also near to the nuclear export signal sequence (40). Normally, IκBα sequesters NF-κB in the cytoplasm and inhibits NF-κB function. When HBx–IκBα form a complex, IκBα is unable to bind and export NF-κB from the nucleus, leading to NF-κB activation. Consequently, the HBx–IκBα complex attenuates NF-κB-induced apoptosis that is mediated by targeting anti-apoptotic genes such as fas, c-FLIP and survivin, or genes from the Bcl-2 family (39). Moreover, Bcl-2 and Bcl-xL also belong to HBx interacting partners.

Recent structural data showed that cellular anti-apoptotic Bcl-xL binds HBx via the BH3-like domain of HBx (41). Crystallographic data demonstrate that the BH3-like motif of HBx interacts with Bcl protein differently than other cellular proteins containing the BH3 domain. Interestingly, the HBV replicon comprising double mutant of HBx (W120A, L123A) failed to produce HBsAg and HBeAg in mice *in vivo*. *In vitro*, isothermal titration calorimetry measurements based on heat exchange recording during HBx titration to Bcl-xL confirmed that the interaction between HBx and Bcl-xL is lost in the W120A, L123A HBx double mutant.

Furthermore, cell survival is promoted because HBx directly binds GRP78, a master regulator that triggers the stress response in the endoplasmic reticulum via the unfolded protein response system. HBx–GRP78 complex reduces ATF4, Bcl-2 and γH2AX expression levels, promotes cell survival and downregulates DNA repair (42). Hence, GRP78 was suggested as a potential therapeutic target for suppressing tumorigenic processes (43).

Another hallmark of stress caused by HBx in tumorous hepatoma cells is the occurrence of shortened telomeres compared to adjacent hepatic tissue. HBx induces telomere shortening through binding the c-myc zing finger of MAZ. The complex HBx–MAZ binds preferentially to the telomerase promoter, suppresses its transcriptional activity and downregulates telomerase expression. Therefore, HBx works as a corepressor of MAZ that enhances telomerase repression (44).

HBx not only plays a vital role in promoting cell survival in HBV-positive cells, but also contributes to HBV-related tumorigenesis. HBx supports the stability of cortactin CTTN and transcription factor PAX8, two proteins accepted as tumor markers overexpressed in various cancers (45). Immunoprecipitation confirmed HBx–CTTN complex formation in HepG2 and metastatic hepatoma MHCC-LM3 cell lines. The CTTN levels correlate with increased levels of CREB1 as well as CREB1 downstream signaling toward tumor promotion and cell invasiveness. The complex HBx–CTTN restores upregulation of CREB1 signaling in CREB1 knockdown (46). The HBx colocalizes with the E2 ubiquitin ligase complex, where it interacts with Skp2. In the presence of the HBx–Skp2 complex, the ubiquitination omits PAX8 degradation. Consequently, the PAX8 level increases and mediates hepatocarcinoma development (45).

Besides the indirect influence on transcription via interaction with other transcription factors, HBx directly interacts with lncRNAs, including lncRNA DLEU2 (deleted in lymphocytic leukemia 2). As a result, HBx enhances DLEU2 transcription and its accumulation in HBV-infected cells and HBV-related hepatocellular carcinomas, serving as a tumorigenesis marker. HBx–DLEU2 interaction sites partially overlap with sites of another DLEU2 interacting partner – histone methyltransferase enhancer of zeste homolog 2 (EZH2), the catalytic subunit of the polycomb repressor complex 2 (PRC2), which belongs to the group of chromatin-modifying complexes. During HBV infection, HBx levels increase. If HBx:EZH2 ratio is 6:1 or higher, DLEU2 favors HBx binding. DLEU2–HBx co-recruitment on the cccDNA displaces EZH2 from the viral chromatin, supporting the transcriptionally active state of viral cccDNA and replication of the whole virus (47).

### Implications and perspectives of HBx regulation in oncogenesis

Current HBV medication relies on the long-term HBV attenuation by a mixture of nucleoside/nucleotide analogs in combination with interferons and their pegylated derivatives, as reviewed recently (48). Entecavir and two derivatives of tenofovir (disoproxil fumarate and alafenamide) are novel antivirals that display better resistance barriers in prolonged administration compared to older drugs. Another nucleotide analog candidate (ATI-2173) acts specifically as an HBV polymerase inhibitor and exhibits improved pharmacokinetics (49). Moreover, by acting in a noncompetitive manner, it is expected to effectively suppress HBV recurrence in combination with other nucleotide/nucleoside analogs.

A promising primary target for antiviral drug design among HBx-binding proteins might be DDB1, as HBx–DDB1 interaction was validated by structural studies (32). Importantly, HBx forms a complex with Cul4–DDB1 ubiquitin E3 ligase, where HBx acts as a ‘substrate adaptor’ via HBx–DDB1 interaction. HBx recruits critical cellular proteins for ubiquitination that ought to be removed to regulate host cell pathways successfully (50). Compromising the complex HBx–DDB1 by interfering molecules may significantly downregulate HBx activity and the whole HBV genome transcription. The attempts to find the inhibitors using a split luciferase assay system have been initiated (51).

Recently, other HBx structural studies identified a short oligopeptide comprising amino acids 118-127 that outcompetes the active motif of viral HBx from binding with Bcl-xL (41). Cellular metabolism impairment mediated via the BH3-like domain of the HBx oligopeptide was demonstrated *in vivo*. On this account, HBx oligopeptide presents a promising way to attenuate HBV infection (41). Coincidentally, the first synthetic BH3 mimetic (venetoclax) was approved by FDA as apoptosis-promoting drug in the treatment of chronic lymphocytic leukemia (52). The current research directed to the personalized profiling of BH3-mediated anti-apoptotic status of tumor cells could lead to the proper BH3 mimetic selection for an effective cure of difficult-to-treat cancers. Additionally, selective HBx oligopeptide treatment could prevent hepatocarcinoma promotion.

Similarly, HBV could be inhibited specifically on multiple levels by disrupting DLEU2–HBx interaction. The impairing of DLEU2 binding would silence cccDNA transcription. Moreover, preventing DLEU2–HBx association with target host promoters would maintain EZH2-mediated repression and lead to the transcriptional silencing of EZH2/PRC2 target genes that would have been regulated via HBx (47). Thus, finding a specific inhibitor disrupting DLEU2–HBx interaction would compromise both transcriptional and epigenetic activation at once.

The reviewed essential and recent findings underscore the importance of a deep understanding of interactions between HBx and cellular partners. The ongoing research exploring small molecule agonists and inhibitors shows encouraging results applicable in the treatment of especially chronic HBV patients with complications. As hepatocarcinoma is an aggressive cancer with high resistance to chemotherapy (53), the therapy based on the HBx-interfering approach is an attractive way to efficiently treat this type of cancer.

As HBx interacts with many host partners, HBV infection treatment based on HBx inhibition might require the combination of several specifically targeted drugs. Furthermore, the comprehensive HBx interface involved in protein–protein interactions, together with utilizing shallow interaction pockets, might present complications in molecular research that could make HBx targeting challenging (54). Despite the possible structural and functional challenges connected to the unique HBx role in HBV infection, the invention of precise and effective HBx targeting deserves the full attention of the whole research community.

### HTLV-1: life cycle and strategy of viral genome transcription

**Figure 3.**
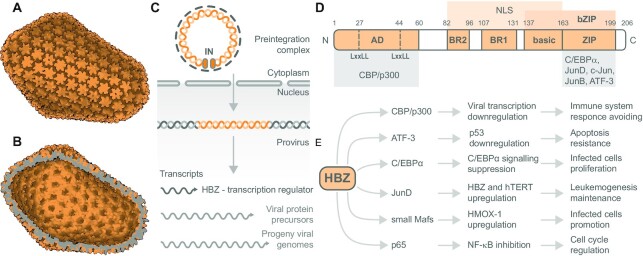
The retroviral capsid, viral RNA processing and HBZ transcription factor domain structure, interactions and effects. The electron microscopy reconstruction of HIV-1 capsid based on PDB 3J3Y: (**A**) outer view and (**B**) inner view, visualized using the Protein Imager. HTLV-1 and HIV-1 are close relatives within the Retroviridae family. (**C**) Viral RNA processing cycle of HTLV-1. The two copies of viral genomic RNA are reverse transcribed to form the pre-integration complex. The reverse-transcribed viral DNA and integrase (IN) enter the nucleus. The viral DNA incorporates into the host genome via integrative recombination catalyzed by IN. Thus, the created provirus is transcribed into mRNA. Expressed mRNA molecules produce viral proteins, including the transcription regulator HBZ and new viral genomic RNA. (**D**) Domain structure of viral transcription factor HBZ – the most abundant isoform. HBZ possesses an N-terminal activation domain (AD) containing two LxxLL motifs interacting with transcription coactivators CBP/p300. Central basic regions (BRs) of HBZ are responsible for nuclear localization. The C-terminal bZIP domain enables HBZ to heterodimerize with several bZIP factors. The bZIP domain consists of the basic region involved in DNA binding and a ZIP that mediates coiled-coil interactions with other bZIP transcription factors. (**E**) Overview of cellular proteins interacting with HBZ along with effects of the interactions on cellular functions and subsequent HTLV-1 progression and propagation. Other HBZ interacting partners are mentioned in the text.

HTLV-1 is a delta RNA retrovirus that was first isolated in the early 1980s by two independent research groups (55,56). HTLV-1 belongs to the Retroviridae family, including the human immunodeficiency virus (HIV) (Figure 3A and B). HTLV-1 infects an estimated 15 million people worldwide annually (57). Although HTLV-1 infection is mostly asymptomatic, ∼4% of carriers develop a severe malignancy of CD4^+^ T cells known as adult T-cell leukemia/lymphoma (ATL) after a latency period of three to six decades (58,59). HTLV-1 is also the causative agent of several inflammatory and immune-mediated diseases, most notably HTLV-1-associated myelopathy/tropical spastic paraparesis (60), and to a lesser extent HTLV-1 uveitis (61), infective dermatitis (62), myositis (63), arthritis (64) and bronchiectasis (65). ATLs are clinically classified into four distinct subtypes: acute, lymphomatous, chronic and smoldering. Acute and lymphomatous ATLs are highly aggressive with a median survival of <1 year, whereas chronic and smoldering represent more indolent forms of the disease (66,67).

Viral RNA processing in HTLV-1 is initiated by viral reverse transcriptase that mediates the synthesis of a dsDNA copy of the viral RNA genome. Viral DNA and integrase protein gain access to the nucleus mainly using intracellular trafficking machinery (Figure 3C). Viral integrase catalyzes viral DNA insertion into the host DNA (23). Transcription of integrated viral DNA (the provirus) by the host cell RNA polymerase II produces full-length RNA transcripts, which are used for multiple purposes: (i) full-length RNA molecules that are exported from the nucleus and serve as mRNAs to form Gag and Gag–Pol polyprotein precursors; (ii) parts of full-length RNA molecules become encapsidated as progeny viral genomes; and (iii) other full-length RNA molecules are spliced within the nucleus to form mRNA for the Env polyprotein precursor proteins (23).

HTLV-1 is transmitted mainly by breastfeeding (68), and to a lesser extent through sexual intercourse (69), and exposure to cell-containing infected blood components through transfusion or needle sharing (70,71). HTLV-1 transmission is mediated by cell-to-cell contact from infected to uninfected cells, not via free virions (72), and utilizes the activity of regulatory and accessory genes encoded by the 3′ pX region (*tax*, *rex*, *p30*, *p12*, *p13* and *HBZ*) of HTLV-1 provirus (73). Rex has been shown to regulate post-transcriptional viral gene expression. Moreover, Rex increases viral RNA stability during the latency phase of the viral life cycle (74,75). The genes *p12*, *p13* and *p30* play significant roles in establishing and maintaining viral persistence (76). HBZ and Tax interact with host proteins to alter their function and manipulate host cell signaling and transcriptional pathways, thus mediating HTLV-1 oncogenesis with successful evasion of the immune surveillance (77). HBZ is significant for the proliferation of infected cells, whereas Tax is crucial for viral replication and *de novo* infection. In contrast to the intermittent expression of Tax in ATL cells, HBZ is consistently expressed and antagonizes several Tax activities (77).

Tax plays an essential role in several mechanisms involved in leukemogenesis mediated by HTLV-1, including down- and upregulation of numerous microRNAs, differential mRNA expression, alternations in cell signaling, somatic mutations, and epigenetic deregulations or aneuploidy (78). Tax also activates several cell signaling pathways such as pathways depending on serum response factor, CREB/ATF (cAMP response element-binding protein/activating transcription factor family protein) and NF-κB. Thus, Tax modulates viral and cellular gene expression (79,80).

Moreover, Tax affects the mechanisms controlling cell cycle progression, DNA damage response and apoptosis that allow Tax to favor the proliferation of infected cells and the genomic instability accumulation (78,80). Additionally, Tax has the potential to transform rodent fibroblasts (81) and induce cell transformation in transgenic mice (82). Unexpectedly, Tax has been reported to rarely transform human primary T cells, suggesting that other viral proteins rather cooperate to induce T-cell transformation and ATL development (83). For example, NF-κB activation mediated by Tax represents a crucial step in the promotion of cell proliferation, which HBZ balances to enable the infected cells to escape immune surveillance (84).

Recently, Vandermeulen *et al.* analyzed the transcriptome and interactome of the Tax and HBZ proteins. They uncovered distinct but common host factors and RNA-binding proteins (such as U2AF2, a critical cellular regulator of pre-mRNA splicing) between HBZ and Tax. The authors found out that Tax and HBZ alter the splicing landscape in T cells. Interestingly, Tax favored exon inclusion, while HBZ induced exon exclusion. A subset of the splicing changes was also found in ATL patients and might represent clinically valuable biomarkers for ATL (85).

### HBZ structure and binding partners: transcription regulation roles

HBZ is a 206-amino acid, 25-kDa protein. Figure 3D shows the predominant spliced transcript in ATL cells that is transcribed from a negative-sense RNA under the control of a 3′ LTR promoter (73). HBZ possesses an N-terminal activation domain containing two LxxLL-like motifs that interact directly with the KIX domain of transcription coactivators CBP/p300 (Figure 3D and E). The KIX domain of CBP/p300 is also recognized by Tax (86). Once HBZ interacts with CBP or p300, HBZ interferes with Tax’s ability to bind CBP/p300. Thus, HBZ prevents CBP/p300 recruitment to the viral promoter and represses Tax-dependent HTLV-1 transcription activation. Moreover, HBZ interacts with other CBP/p300 domains, including both the HAT and C/H3 domains (87). Therefore, HBZ inhibits HAT activity of p300/CBP, causing a reduction in p53 acetylation and repression of p53 activity (88). HBZ also contains three distinct regions in its central part that direct HBZ to the cell nucleus (Figure 3D): two nuclear localization signals (NLSs) correspond to basic regions containing the stretch of three to five positively charged residues, known to be present in several NLSs of nuclear proteins. The third basic region precedes a leucine zipper domain (ZIP) located at the C-terminal region of HBZ (Figure 3D). Together, the C-terminal regions form a bZIP domain that regulates the activity of several bZIP factors (Figure 3D and E). ZIP forms coiled-coil interactions with similar domains found in other bZIP transcription factors such as CREB, CREB-2, ATF1, ATF2, ATF3, C/EBPα and c-Jun. Consequently, heterodimerization between HBZ and these factors affects positively or negatively their transcription (Figure 1C).

Specifically, HBZ inhibits C/EBPα and c-Jun activity by sequestering them into transcriptionally inactive nuclear bodies, which supports the proliferation of ATL cells (89,90). Additionally, HBZ interacts with JunD (Figure 3D and E), a transcription factor, that is highly expressed in ATL cells. Heterodimerization of HBZ with JunD stimulates JunD expression from the viral 3′ LTR and enhances transcription of cellular genes such as *hTERT*. Consequently, HBZ reactivates telomerase and therefore may contribute to the development and maintenance of the leukemic process (89,90). HBZ has also been reported to activate transcription of*HMOX1*, an oxidative stress response gene, via the interaction with small Maf proteins – transcription factors MafF, MafG and MafK (Figure 3D and E) (91). The increased levels of HMOX1 by HBZ protect cells from oxidative stress. Therefore, HBZ supports ATL maintenance.

Due to constant HBZ expression in infected cells, HBZ is thought to be essential for the differentiation of infected cells into various hematopoietic cells. To enable HTLV-1 transmission and to evade host immune response, HBZ promotes transcription of Foxp3, CCR4 and T-cell immunoreceptor with Ig and ITIM domains (TIGIT) (77).

Additionally, HBZ suppresses host classical NF-κB-driven transcription mediated by p65 (Figure 3E) by two mechanisms: (i) the inhibition of p65 DNA binding and (ii) the enhanced degradation of p65 through PDLIM2 E3 ubiquitin ligase expression. Moreover, HBZ represses transcription of some classical NF-κB target genes, including IL8, IL2RA, IRF4, VCAM1 and VEGFA (92). A typical example of the NF-κB target gene that HBZ regulates is the cyclin D1 promoter gene. Cyclins control cyclin-dependent kinases and thus regulate the G1/S phase transition of the cell cycle. Ma *et al.* found that HBZ inhibits cyclin D1 mRNA level through binding with CREB, an action opposite to the G1/S transition induced by Tax (93). These findings suggest that the HBZ-mediated inhibition of NF-κB in cooperation with Tax-mediated activation is beneficial for infected cells’ proliferation and oncogenesis.

Interestingly, not only HBZ protein but also HBZ RNA, which cannot produce the HBZ protein, is implicated in cell proliferation and ATL (94). HBZ RNA enhances transcription of the survivin gene, which counteracts apoptosis induction by HBZ protein. In this way, HBZ protein and HBZ RNA can oppose one another. The RNA–protein competition may present an elegant mechanism for controlling the proliferation and survival of ATL cells and HTLV-1-infected cells.

### Implications and perspectives of HBZ regulation in oncogenesis

ATL treatment is highly challenging due to the short median survival of patients diagnosed with aggressive forms (12). Moreover, HTLV-1 can avoid immune detection while maintaining or increasing the viral reservoir, which makes the treatment and prevention difficult and should be considered when developing future strategies (73).

The current treatment of ATL includes multi-agent chemotherapy (67) or allogeneic hematopoietic stem cell transplantation (95). Nowadays, patients with ATL lymphoma rely on chemotherapy with concurrent or sequential antiretroviral therapies consisting of zidovudine (ZDV) and IFN-α combination (96). Eventually, aggressive forms of ATL are treated by chemotherapy combined with humanized anti-CCR4 monoclonal antibodies (95). Intensive research in the last several years brought novel ATL treatment approaches, including pro-apoptotic molecules such as Bcl-2 inhibitors (95), cyclin-dependent kinase 9 inhibitors (97), and combined arsenic/interferon therapy with ZDV and IFN-α (98). In more detail, the promising mechanisms and drugs developed to inhibit ATL cell proliferation or to induce apoptosis have been recently reviewed elsewhere (78,99,100).

With millions affected worldwide, HTLV-1 is a significant problem in endemic communities. Remarkably, there are no effective vaccine or treatment options to prevent ATL so far. Current clinical treatments can slightly improve the overall patient survival (101). However, ATL patients’ median survival is still <1 year. To help identify and develop novel effective therapies for ATL, we need a deeper understanding of molecular events leading to HTLV-1-induced oncogenicity. We expect that discovering both novel preventive or therapeutic strategies for HTLV-1 treatment might bring relief to infected individuals. Moreover, the new approaches could augment the understanding of other infections with oncoviruses.

As the HTLV-1-mediated oncogenesis is a cumulative result of multiple proteins, there can be several strategies used to prevent and treat ATL. Based on the successful entry inhibition of another retrovirus, the HIV (102), previous approaches to treat HTLV-1 treatment have focused on the entry steps of HTLV-1 replication cycle since binding of the virus to the cellular entry factors is required for viral transmission to non-infected cells. The strategies targeted envelope glycoprotein (Env) as a potential candidate for HTLV-1 vaccine and therapeutics (103). Simultaneous targeting of Env and accessory proteins including Tax and HBZ (80,104) could increase treatment efficiency and improve recovery prognosis.

The Tax sensitivity to host immunity and ubiquitous HBZ expression in ATL cells makes these viral proteins attractive therapeutic targets (105). For example, a complex and cooperative interplay between Tax and HBZ maintains the NF-κB signaling equilibrium to drive clonal expansion balanced by senescence inhibition. The malignant transformation of infected T cells likely depends on the optimal level of signal transduction fine-tuned by Tax and HBZ (80). It is critical to thoroughly elucidate the relation between Tax and HBZ for a deeper understanding of oncogenic mechanism mediated by HTLV-1.

There has been no vaccine preventing HTLV-1 confirmed in clinical trials yet. Recently, an anti-HTLV-1 lentiviral vector-based vaccine that encodes a unique polypeptide derived from Tax, HBZ, p12 and p30 accessory viral proteins has been developed. Experiments in mice have proven that the vaccination is safe and efficient inducing a cellular response that makes the vaccine promising for further studies (78). Additionally, Raza *et al.* carried out *in silico* studies that suggested the development of a vaccine against HTLV-1 vTRs. The authors predicted HBZ and Tax antigenicity and concluded that HBZ is a potential drug target. In contrast, Tax is a potential candidate for an epitope-based universal vaccine, which might activate the acquired immune system and stimulate the desired immune response against HTLV-1 (103). However, the vaccine potency should be confirmed via *in vitro* and *in vivo* immunological assays.

As HBZ performs an essential role in the proliferation of HTLV-1-infected cells, HBZ might provide a unique unrevealed mechanism that allows infected cells to evade immune recognition (106). One of the open questions is whether there is a direct connection between HBZ and cell energy deregulation, as the only missing cancer hallmark associated with HBZ. Direct HBZ connection with all essential cancer characteristics *in vivo* is yet to be confirmed to show a specific HBZ role in oncogenesis and to prove HBZ as an effective target for the development of new anti-HTLV-1 therapies.

Despite the growing evidence that HBZ is implicated in HTLV-1 pathogenesis, there is still little known about HBZ RNA functions. According to Gazon *et al.*, HBZ RNA inhibits sense transcription of HTLV-1 (107). They observed an RNA-dependent mechanism leading to a complete silencing of viral expression. Consequently, the induced viral expression silencing allows HTLV-1 entry into latency and escape from the immune response. Inhibition of HBZ RNA function in the mechanism could present a potential strategy for HTLV-1-associated disease treatment. Taken together, we suggest that a deeper understanding of HBZ functions might indicate new perspectives for the regulation of HTLV-1 progress and subsequent potential clinical applications.

### EBV: life cycle and strategy of viral genome transcription

**Figure 4.**
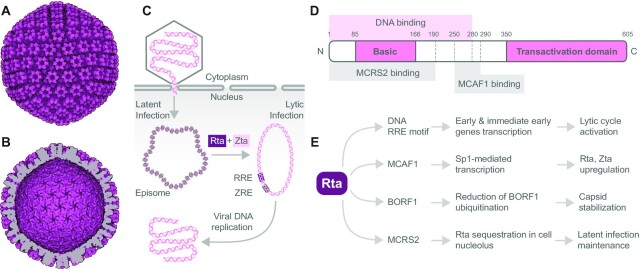
EBV capsid, viral DNA processing, and Rta transcription factor domain structure, interactions and effects. The electron microscopy reconstruction of EBV capsid particle based on PDB 7BSI: (**A**) outer view and (**B**) inner view, visualized using the Protein Imager. (**C**) Schematic representation of how EBV genome is processed in the host cell. Transcription factors Rta and Zta induce transformation of the latent to lytic infection of EBV. Rta and Zta bind to their response elements and converse the episomal viral DNA condensed on histones to the replication-ready circular dsDNA to produce viral DNA. RRE, Rta response element; ZRE, Zta response element. (**D**) Domain structure of viral transcription factor Rta. Rta contains the N-terminal basic and C-terminal transactivation domain. DNA binding region is localized within the 280 N-terminal amino acids. The Rta amino acid region 250-290 is essential for MCAF1 binding, important for Rta upregulation. The interaction with MCRS2 through Rta amino acid region 1-190 sequesters Rta in the nucleolus. (**E**) Overview of viral and cellular proteins interacting with Rta along with effects of the interactions on viral and cellular functions and subsequent EBV progression and propagation.

EBV is a ubiquitous human virus that belongs to the gamma-herpesvirus family. EBV was initially isolated from a cultured Burkitt’s lymphoma (BL) cell line by Epstein *et al.* in 1964 (108). In 1984, EBV became the first fully sequenced herpesvirus with 172 kb genome encoding over 80 genes. The viral genome includes Epstein–Barr nuclear antigens (EBNAs) and latent membrane proteins (LMPs). The EBV genome persists in linear dsDNA form in viral particles (Figure 4A and B). DNA circularization is achieved by joining multiple 0.5 kb long terminal direct sequence repeats, creating the so-called episome (Figure 4C). The internal sequence repeats divide the viral genome into long and short unique domain regions (109). EBV infects >90% of the human population during their lifetime, mostly without developing any symptoms (110). EBV spreads primarily through saliva. Studies have proven that EBV is a primary cause of infectious mononucleosis (111). Besides infectious mononucleosis, 200 000 cancer cases per year are also linked to EBV (112). Moreover, EBV is also associated with the development of autoimmune diseases such as multiple sclerosis (113).

EBV mainly infects two cell types: B lymphocytes and epithelial cells. Less commonly, EBV can infect natural killer T cells. EBV-related cancers differ based on the infected cell type (114). When EBV targets B lymphocytes, Hodgkin’s lymphoma, BL or post-transplant lymphoproliferative disorder (PTLD) can develop. If EBV infects epithelial cells, EBV can cause nasopharyngeal carcinoma (NPC), gastric carcinoma (GC) or breast cancer (BC) (115).

EBV infection occurs in two states: latent and lytic (Figure 4C). EBV commonly infects B lymphocytes or epithelial cells in latent form and uses the host’s polymerases to replicate during cell division. On one hand, a minimal number of viral genes are expressed in the latent state (116). On the other hand, Epstein**–**Barr encoded RNA, a small noncoding and non-polyadenylated RNA, is highly expressed in the latent state (117) and can be used as a marker of EBV infection (117,118). The latent state allows long-term survival of the EBV in human host cells where the viral genome persists as an episome in the nucleus (Figure 4C). The latent genes’ expression can be involved in cancer development. For instance, EBNA-1, one of the latent genes, plays a role in BL, and LMP-1 is involved in NPC. The transition from latent to lytic state is called virus reactivation. *In vitro*, the reactivation is triggered by 12-*O*-tetradecanoylphorbol-13-acetate, sodium butyrate, anti-Ig and TGF-β (transforming growth factor-beta). Nevertheless, the exact molecular mechanism that drives reactivation *in vivo* remains enigmatic (119).

It was shown that immediate early genes *BRLF1* and *BZLF1* that encode transcription factors Rta and Zta, respectively, play an essential role in virus reactivation (120). Rta and Zta upregulation is necessary for full expression of the EBV proteins, including viral DNA polymerases, during the immediate-early stage of the lytic cycle (121). Rta can also upregulate its own expression via a positive autoregulatory loop (Figure 1B) (122). Significantly, Zta closely cooperates with Rta to synergistically activate EBV early genes. Zta has been reported as the first transcriptional factor that preferentially binds to and activates the methylated *BRLF1* promoter (Rp). Zta binding to the methylated Rp, facilitated by serine residue 183, activates transcription of Rta (123,124). Thus, the binding of Zta to Rp can serve as a switching mechanism between the latent and lytic states of the EBV infection (Figure 4C).

Zta belongs to the family of basic leucine zipper (bZIP) transcription factors with a transactivation domain that was described to be essential for recognition and high-affinity binding to methylated DNA (125). Additionally, Zta can be secreted from the cell and directly penetrate the cell membrane using its cell-penetrating domain. In EBV-infected cells, Zta alone can switch EBV from latency to lytic cycle. Therefore, Zta may transfer reactivation signals between infected cells (126,127). During the latent stage of the virus cycle, the EBV virion genome becomes heavily methylated by the cellular DNA methylation machinery (128). Zta binds methylated DNA and activates transcriptionally silent host genes (129,130). It was suggested that *BZLF1* gene expression could directly or indirectly contribute to EBV-induced tumorigenesis. Zta involvement in EBV-induced oncogenesis was recently reviewed comprehensively (131).

### Rta structure and binding partners: transcription regulation roles

Rta is a 605-amino acid, 67-kDa protein encoded by the EBV gene *BRLF1* (Figure 4D). Histone acetylation at the BRLF1 promoter allows Rta expression, which further leads to activation of the viral lytic cycle (132). Rta is one of the least structurally characterized vTRs. It was shown that Rta forms homodimers but not monomers in solution. DNA binding region of Rta is localized within the 280 N-terminal amino acids and the dimerization region within the 232 N-terminal amino acids. Furthermore, no direct homologies were identified compared to other known DNA binding or dimerization motifs. The C-terminal part of Rta contains a transactivation domain rich in proline and acidic residues (133).

Rta presents a vTR of EBV that directly binds to the Rta response element (RRE) and transactivates a series of lytic genes, including the viral lytic gene PAN (Figure 4D and E). Rta binding to RRE has been described qualitatively by EMSA (134). Moreover, the Rta mutant lacking the C-terminal part comprising the last 55 amino acids can still bind DNA and display binding activity to BMLF1 promoter *in vitro* (135). Importantly, Rta can also form a complex with other proteins and regulate transcription via a mechanism independent of the RRE binding (136).

As mentioned before, the synergistic activity of Rta with Zta is a crucial factor in activating the series of early lytic genes. It was shown that Rta interacts with Zta through MCAF1 (MBD1-containing chromatin-associated factor 1) (Figure 4E). The assembled complex binds to Zta response element (ZRE) and synergistically promotes the transcription activation of the EBV lytic genes (137). MCAF1 plays a critical role in the AP-1-dependent Rta activation of BZLF1 transcription due to MCAF1 complex formation with Rta and ATF2. Moreover, the Rta–MCAF1–ATF2 complex binds the BMRF2 promoter, a critical viral gene for EBV infection (138). The Rta amino acid region 255-290 is essential for MCAF1 binding (Figure 4D). MCAF1 also mediates Rta interaction with Sp1-interacting protein, where Rta increases the Sp1-mediated transcription, leading to the upregulation of Rta and Zta (139).

Recently, it was found that Rta is stably expressed in B lymphocytes during EBV latency and is present mainly in the nucleolus. The nucleolar sequestration of Rta is regulated by the interaction with MCRS2 protein (Figure 4D and E) and impacts the EBV lytic progression by inhibiting the Rta transactivation activity. Additionally, Rta is associated with ribosomes and enhances cellular translation in the nucleolus (140).

Another essential feature of Rta is initiating a cellular senescence program in epithelial cells and promoting the autophagy induction via ERK1/2 activation in the early lytic phase. Taken together, Rta can stimulate growth arrest, induce senescence and modulate autophagy in infected cells (36,122).

Despite the Rta function as a regulator of host factors, it can also interact with viral proteins. Specifically, the Rta is present on the EBV capsid, where it directly interacts with EBV capsid protein BORF1 (Figure 4E). Rta reduces ubiquitination and thus stabilizes BORF1. Hence, Rta acts as an EBV tegument protein that stabilizes EBV viral capsids (139).

### Implications and perspectives of Rta regulation in oncogenesis

EBV-related oncogenic diseases are often fast-growing tumors where early detection and immediate treatment are crucial for successful recovery. Regardless of the relatively high cure rate of B lymphocyte malignancies in developed countries, the cure rate remains low in less developed countries where health care is less available. In contrast, epithelial cell malignancies such as NPC, GC or BC have overall high mortality. Mechanisms of EBV-related oncogenesis have been already thoroughly reviewed elsewhere (141–144).

Despite the progress that has been made in the comprehension of the EBV connections to cancers, several aspects of EBV-related oncogenesis are still unknown and represent a major challenge in cancer research. Thus, new and potentially less demanding treatments, not involving chemotherapy and radiotherapy, that target EBV latent genes expression and vTRs could increase treatment efficiency and improve recovery prognosis (141,142).

EBV is associated with several malignancies, including PTLD. PTLD treatment consists of rituximab and immunosuppression reduction together with chemotherapy (145). Cellular therapy utilizing EBV-specific cytotoxic T lymphocytes provides an alternative to the conventional treatments of PTLD (146). Another strongly driven malignancy by EBV is NPC. The EBV protein LMP-1 was identified as a potential therapeutic target against EBV-positive NPC. LMP-1 promotes tumorigenesis by affecting various oncogenic pathways (147). Recently, DNAzyme (DZ1) was designed to target the LMP-1 mRNA specifically. DZ1-based treatment could reverse the malignancy and promote the radiosensitivity of NPC (148).

The lytic form of EBV can be effectively inhibited by the guanine nucleoside analogs acyclovir and ganciclovir. They both inhibit viral DNA replication by silencing the viral DNA polymerase during the lytic cycle (149,150).

The majority of EBV-associated diseases are indirect results of viral replication. Moreover, EBV-associated malignancies are caused by the expression of viral genes that function as survival signals keeping EBV in a latent state. Therefore, inhibiting viral replication is ineffective in treating EBV-associated malignancies or infectious mononucleosis. As an alternative approach, the lytic cycle induction has been proposed as a therapy. Adenovirus vectors expressing Rta and Zta can be used to stimulate viral replication via lytic cycle activation (151), thus sensitizing EBV-infected tumor cells to nucleoside analogs. Combined treatments using analogs and chemotherapy are more effective than monotherapy (152,153). Even though the lytic cycle can inhibit tumorigenesis due to the final lysis of the infected cells, several lines of evidence suggest that lytic cycle initiation contributes to tumor progression (143,154). Since Rta and Zta induce the lytic cycle in cooperation with other viral and cellular proteins, Rta and Zta could be employed to prevent EBV-driven oncogenesis after lytic cycle initiation. For example, targeting EBV replication by antirheumatic drug leflunomide inhibits the earliest step of lytic EBV activation – *BZLF1* and *BMRF1* expression and prevents the progress of EBV-induced lymphomas (155).

The complex interactions between the EBV and their hosts have made it challenging to design useful vaccine strategies to protect against EBV-associated diseases (156), and despite more than four decades of development, no licensed prophylactic vaccine against EBV is available (157).

The research has mainly focused on the EBV membrane antigen containing glycoprotein gp350, the uppermost abundant glycoprotein on the surface of virions and the most expressed by cells infected with EBV, as the most suitable vaccine candidate (157). The gp350 vaccine reduces the incidence of infectious mononucleosis. However, in phase II of clinical trials, the vaccine failed to prevent EBV infection. Moreover, designing a live-attenuated herpesvirus vaccine is improbable due to the persistence in infected individuals.

According to the challenges in current EBV treatment, the viral transcription factors present a new potential strategy for therapy. Here, Rta and Zta have been reviewed as essential and indispensable parts of EBV lytic cycle reactivation. Most of the EBV-associated diseases and malignancies develop in the latent viral state. The activation of the lytic cycle sensitizes infected cells to antiviral medication and may prevent the progression of EBV-associated diseases. Hence, the upregulation of Rta presents a promising target for lytic cycle induction as an alternative EBV treatment.

We can hypothesize several approaches for Rta upregulation that could lead to EBV lytic cycle activation: (i) inhibit the Rta–MCRS2 interaction, which could prevent Rta sequestration in the nucleolus and promote the Rta transactivation activity; (ii) design agents that could specifically bind to BRLF1 promoter and activate the Rta translation; (iii) employ adenovirus vectors expressing BRLF1; or (iv) a combination of mentioned strategies. Another approach how to suppress EBV tumors can employ the cytotoxic T lymphocyte’s response to Rta and Zta. Further studies of Rta and its viral transcriptional activity are needed for a deeper understanding of how vTRs could be used for effective EBV treatment.

## RELEVANCE AND PROSPECTS

The complete eradication of virus-induced diseases remains beyond the reach of current therapies despite significant advances in the development of effective vaccines. A surprising challenge is convincing the population that the vaccination is necessary and beneficial, as shown during the COVID-19 pandemic. When the part of the population refuses vaccination, finding the proper antiviral treatment for patients with severe disease outbreaks is even more critical. Thus, the demand for antiviral treatment that might selectively eliminate viral proteins and their activity is urgent.

For the successful spread of infection, viruses have evolved to sneak through the innate and adaptive antiviral response at both the cellular and whole organism levels. The current extensive studies of the life cycle of viruses brought novel molecular and structural knowledge of infection mechanisms. Nevertheless, little is still known about the functions of vTRs that enable viral genes to express and affect host gene expression. As vTRs are critical for effective viral infection, they are central to human disease pathogenesis and attractive targets for therapy.

Nowadays, the emphasis is placed on designing the pan-antiviral agents that could be utilized as a broad-spectrum viral treatment. Here, vTRs present a potential pan-antiviral target, affecting and modulating the host gene signaling pathways. vTRs directly bind specific DNA motifs (Rta and Zta) or protein domains (HBx and HBZ) (18). We can employ a transcription factor decoy (TFD) strategy to design oligonucleotides and oligopeptides to saturate and inactivate the binding sites of vTRs.

Targeting vTRs inside the cell is significantly more problematic as interfering molecules (Figure 1D) have to pass through cell and nuclear membranes. This limit could be diminished by the application of sophisticated drug delivery ways such as improved lipid-based nanoparticles that can deliver a therapeutic agent into the cell and biodegradable vectors designed to deliver mRNA coding protein parts for TFD to the cytoplasm (158). The second challenge is the limited use of antibody-based therapy against vTRs that is connected with the location of vTRs in the nucleus. This challenge can be partially overcome for vTRs that induce antigens on the cell surface. The third challenge is connected with the wide functional and interactional diversity of vTRs. The binding variety of vTRs might bring an interaction detour that would overpass a drug-inhibited interaction. Inhibition of more interacting domains of vTRs would minimize the interaction bypass possibility. The final problem is the long latency time for oncoviruses HTLV-1 and EBV. One of the most promising solutions might be controlled induction of transition from latent to lytic state with subsequent immunotherapy.

The previously mentioned limitations and challenges of vTR targeted treatment are prevailed by high specificity of the therapy focused on viral proteins with expected minimal effects on host cell proteins. In parallel, additional studies of vTRs and their interaction partners should be carried out to identify critical sites for disturbing vTR complexes that have been reviewed here. We suggest that vTRs should be examined by combining advanced methodological approaches *in vitro* and *in vivo* to reveal critical turning points that could be utilized for specifically targeted interference therapy.

The question of how vTRs’ binding to human transcriptional cofactors in host cells affect functions of the human proteins is essential regarding the possible side effects and acquired resistance of inhibiting vTRs. Additionally, what are the most specific and reliable ways of targeting vTRs in relevant cell types? Another cardinal question is what the fate of vTR complexes would be after disabling critical interactions and how it would affect host cells’ response to viral genome expression. Finally, could vTR complexes be disturbed effectively by small molecules and thus used for specific eradication of viral infection? The lack of answers to the questions outlined above hampers the struggle with finding the way to defeat the most common human viruses. Elucidating the mechanism of vTRs’ interaction might shed new light on virology and cell biology and reveal new, more successful antiviral-anticancer therapies. Eventually, revealing the Achilles heel in vTR action will help to develop a new effective treatment for virus-induced diseases against which vaccination strategy could not be applied.
